# Dual Localized AtHscB Involved in Iron Sulfur Protein Biogenesis in Arabidopsis

**DOI:** 10.1371/journal.pone.0007662

**Published:** 2009-10-29

**Authors:** Xiang Ming Xu, Hong Lin, Maita Latijnhouwers, Simon Geir Møller

**Affiliations:** Center of Organelle Research, Faculty of Science and Technology, University of Stavanger, Stavanger, Norway; University of Melbourne, Australia

## Abstract

**Background:**

Iron-sulfur clusters are ubiquitous structures which act as prosthetic groups for numerous proteins involved in several fundamental biological processes including respiration and photosynthesis. Although simple in structure both the assembly and insertion of clusters into apoproteins requires complex biochemical pathways involving a diverse set of proteins. In yeast, the J-type chaperone Jac1 plays a key role in the biogenesis of iron sulfur clusters in mitochondria.

**Methodology/Principal Findings:**

In this study we demonstrate that AtHscB from Arabidopsis can rescue the *Jac1* yeast knockout mutant suggesting a role for AtHscB in iron sulfur protein biogenesis in plants. In contrast to mitochondrial Jac1, AtHscB localizes to both mitochondria and the cytosol. AtHscB interacts with AtIscU1, an Isu-like scaffold protein involved in iron-sulfur cluster biogenesis, and through this interaction AtIscU1 is most probably retained in the cytosol. The chaperone AtHscA can functionally complement the yeast *Ssq1*knockout mutant and its ATPase activity is enhanced by AtHscB and AtIscU1. Interestingly, AtHscA is also localized in both mitochondria and the cytosol. Furthermore, *AtHscB* is highly expressed in anthers and trichomes and an *AtHscB* T-DNA insertion mutant shows reduced seed set, a waxless phenotype and inappropriate trichome development as well as dramatically reduced activities of the iron-sulfur enzymes aconitase and succinate dehydrogenase.

**Conclusions:**

Our data suggest that AtHscB together with AtHscA and AtIscU1 plays an important role in the biogenesis of iron-sulfur proteins in both mitochondria and the cytosol.

## Introduction

Iron-sulfur clusters ([Fe–S]) are important prosthetic groups of iron-sulfur proteins involved in numerous vital biological processes, such as respiration, photosynthesis and nitrogen fixation, and inappropriate cluster formation has detrimental consequences in both prokaryotes and eukaryotes [1]. Although simple in structure, [Fe–S] are not formed *de novo* but require an intricate interplay of highly specialized proteins and both genetic and biochemical studies have identified several pathways for the biogenesis and maturation of [Fe-S] in bacteria, yeast and humans [2]–[13], [14], [15]. Numerous diseases, including several neurodegenerative and hematological disorders, have been associated with defects in iron-sulfur protein biogenesis [13], [15].

Iron-sulfur proteins were first identified in plants and several excellent reviews have summarized both early and recent updates in the field of plant [Fe-S] biogenesis [16]–[20]. Most research has been performed on the model plant *Arabidopsis thaliana* where the spatial localisation of different [Fe-S] biogenesis pathways in different subcellular compartments has received increased attention. Besides harbouring a bacterial SUF-like (mobilization of sulfur) system encompassing the SufA, SufB, SufC, SufD, SufS and SufE proteins [21]–[30], chloroplasts also harbour several other components such as Nfu-like proteins [31]–[34], HCF101 [35], APO1 [36] and monothiol glutaredoxins [37]. SufA-like and Nfu-like components and monothiol glutaredoxins are proposed to act as scaffold proteins for [Fe-S] biosynthesis [21], [30]–[34] whilst SufS- and SufE-like components act as a cysteins desulfurase complex [20], [23], [24], [27], [28], [38] extracting sulfur from the amino acid cysteine. SufB-, SufC- and SufD-like elements can form a protein complex which may provide energy through ATP hydrolysis however, the exact function of the SufB/SufC/SufD complex is not clear [22], [25], [26]. Indeed the relationship between the SUF-like system, the Nfu-like components, HCF101, APO1 and monothiol glutaredoxins is unknown in plants.

Compared to chloroplasts, [Fe-S] biogenesis in plant mitochondria has attracted much less attention. Kushir *et al* pioneered this field by identifying the Arabidopsis Sta1 as an Atm1p-like ABC transporter of yeast supporting the maturation of [Fe-S] protein in mitochondria [39]. Further efforts have now identified several components in plant mitochondria that are evolutionarily conserved and similar to that of the yeast ISC-like (iron sulfur cluster) system [31], [40], [41]. In this context it is worth mentioning that the SufE-like protein AtSufE is localized to both mitochondria and chloroplasts where it activates both mitochondrial and chloroplastic cysteine desulfurase [27] indicating a possible spatial link between [Fe-S] biogenesis systems in Arabidopsis [42].

In the plant cytosol, [Fe-S] biogenesis is much less well understood. However, recent work by Balk and colleagues [43] has started to unravel cytosolic [Fe-S] biogenesis. They report that AtNBP35, similar to the NBP35 protein which is part of the cytosolic Cfd1-Nbp35 complex mediating Fe-S cluster assembly in yeast [12], has retained similar Fe-S cluster binding and transfer properties and performs an essential function [43]. However, much work is still required in order to assemble a model of [Fe-S] biogenesis in the plant cytosol.

In bacteria and yeast, the HscA/Ssq1 chaperones and the HscB/Jac1 co-chaperones are important elements of the ISC-like system. HscA/Ssq1 are ATPases, stimulated by the J-type co-chaperone HscB/Jac1 and have been shown to interact with the scaffold protein IscU/Isu, which is regulated by HscB/Jac1 by binding to IscU/Isu to assist [Fe-S] delivery to the chaperone [12], [44]. Yeast Jac1, Ssq1 and Isu have been confirmed to be mitochondrial proteins [12].

Here we demonstrate that Arabidopsis contains a functional AtHscA1/AtHscB/AtIscU1 protein cluster involved in [Fe-S] protein biogenesis. In contrast to yeast, the AtHscA1/AtHscB/AtIscU1 protein cluster is localized to both mitochondria and the cytosol of Arabidopsis suggesting a dual action between these two spatially separate compartments.

## Results

### AtHscB can rescue yeast Jac1knockout mutant

A full-length cDNA (759 nt) encoding the At5g06410 open reading frame was cloned and its predicted amino acid sequence compared to *E. coli* HscB and yeast Jac1 showing 30% and 24% identity, respectively (Figure S1A). The At5g06410 amino acid sequence contains the HPD motif, essential for Jac1 function in yeast [45] and a predicted 59 amino acid N-terminal mitochondrial targeting peptide according to the CBS Prediction Server [46], [47]. As the name Jac1 in Arabidopsis has been assigned to another protein we named At5g06410 AtHscB.

**Figure 1:**
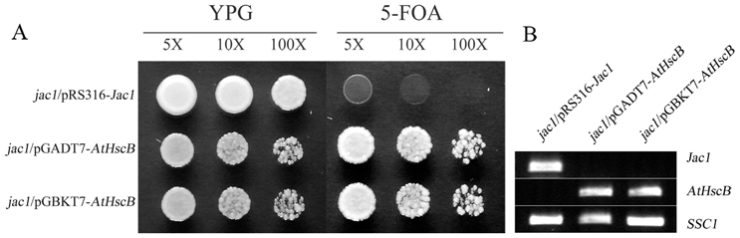
Functional complementation of *Jac1* mutant by *AtHscB*. (A) Three strains, Δ*jac1*/pRS316-Jac1, Δ*jac1*/pGADT7-AtHscB and Δ*jac1*/pGBKT7-AtHscB, were grown overnight in YPD medium and adjusted to similar OD_600_ value. Five-fold serial dilutions were then spotted on a YPD agar plate or on a 5-FOA plate (YPD containing 1 mg/ml 5-FOA). (B) Confirmation of three strains by RT-PCR. First row used *Jac1-*specific primer pair Jac1-L/R; Second row used *AtHscB*-specific primer pair AtHscB-L/R; Third row used *SSC1*-specific primer pair Ssc1-L/R as a control. (Primer sequences can be found on Table S1).

To confirm that AtHscB is a functional homolog of the yeast Jac1 protein, we performed complementation experiments using the lethal yeast knockout mutant Δ*jac1* (Figure 1) [45]. We transformed Δ*jac1*, containing a wild type *Jac1* cDNA on the URA (*URA3*) marked plasmid pRS316, with pGADT7-AtHscB or pGBKT7-AtHscB and positive transformants were screened on synthetic dropout media SD/-Leu (minus L-leucine) or on SD/-Trp (minus L-trypothan), respectively. Once scored the wild-type *Jac1* cDNA was removed by streaking Δ*jac1*/pGADT7-AtHscB and Δ*jac1*/pGBKT7-AtHscB colonies onto YPD media containing 1 mg/ml 5-FOA (5-Fluoroorotic Acid). As *URA3* encodes orotine-5′-monophosphate dicarboxylase, which convert 5-FOA to toxic fluorodeoxyuridine, colonies will only grow where pRS316 has been removed and functional complementation has occurred. On YPD media, Δ*jac1*/pRS316-Jac1, Δ*jac1*/pGADT7- AtHscB and Δ*jac1*/pGBKT7-AtHscB showed clear growth (Figure 1A). However, on media containing 5-FOA Δ*jac1*/pRS316-Jac1 showed no growth as expected whilst Δ*jac1*/pGADT7-AtHscB and Δ*jac1*/pGBKT7-AtHscB grew as well as on YPD media (Figure 1A). RT-PCR confirmed that the two transformed mutants, Δ*jac1*/pGADT7-AtHscB and Δ*jac1*/pGBKT7-AtHscB, had lost wild-type *Jac1* expression but gained *AtHscB* expression whilst the non-transformed mutant, Δ*jac1*/pRS316-Jac1, showed *Jac1* expression (Figure 1B). Combined these results demonstrate that AtHscB is a functional Jac1-like protein.

### AtHscB and AtHscA1 are localized to both mitochondria and the cytosol

**Figure 2:**
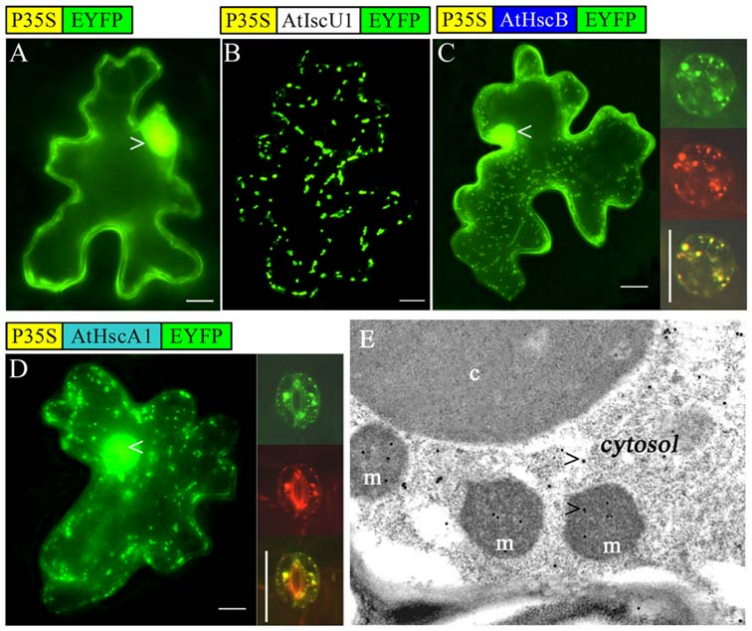
Subcellular localization of AtHscB and AtHscA1. (A) Transient expression in tobacco leaves of pWEN18 (35S-YFP) as a control showing universal localization (Bar for A–D: 50 µm). (B) Transient expression of AtIscU1-YFP showing AtIscU1 localization in mitochondria. (C) Transient expression of AtHscB-YFP in tobacco leaves showing AtHscB localization in both mitochondria and the cytosol. Open arrow indicates the nucleus. The lower panel shows AtHscB-YFP (left), mitotracker dying and merged figure of a stoma from an Arabidopsis seedling transformed with pBA002-AtHscB-YFP. (D) Transient expression of AtHscA1-YFP in tobacco leaves showing AtHscA1 localization to both mitochondria and the cytosol. Open arrow indicates the nucleus. The lower panel shows AtHscA1-YFP (left), mitotracker dying and merged figure of a stoma from an Arabidopsis seedling transformed with pBA002-AtHscA1-YFP. (E) Immunogold labeling and electron microscopy of endogenous AtHscB in Arabidopsis using an AtHscB-specific antibody. Open arrows indicates the gold particles. c = chloroplast; m = mitochondria. (Bar = 2 µm).

To analyze the subcellular localization of AtHscB we fused *AtHscB* to the N-terminus of *YFP* (*Yellow Fluorescence Protein*) and transiently expressed this transgene in tobacco cells (Figure 2). As expected, based on the predicted mitochondrial targeting signal (Figure S1A), fluorescence was observed in mitochondria (Figure 2C). To verify that the fluorescent signal observed was indeed mitochondrial we performed mitotracker experiments which showed that the red fluorescence of mitotracker colocalized with the YFP signal (Figure 2C). However, AtHscB-derived YFP fluorescence was also observed in the cytosol (Figure 2C). To verify that the observed AtHscB dual localization also occurs in Arabidopsis we expressed the AtHscB-YFP fusion protein in transgenic Arabidopsis plants with identical results (Figure S1B). To further confirm the dual localization pattern of endogenous AtHscB protein, we performed immunogold labeling experiments in wild-type Arabidopsis leaves using electron microscopy and an anti-AtHscB antibody. Figure 2E shows the presence of gold particles in both mitochondria and the cytosol strengthening the observed dual localization of AtHscB.

To verify the specificity of the anti-AtHscB antibody we performed western blot analysis of total cell extract from *E. coli* expressing AtHscB and from wild-type Arabidopsis showing the presence of one protein band (Figure S1D). In addition, we performed immunogold labeling experiments using the pre-immune serum showing no signal (Figure S1E).

In Arabidopsis there are two *HscA/Ssq1*-like genes (At4g37910 and At5g09590) whose products are predicted to be mitochondrial (Figure S2) and we named them *AtHscA1* and *AtHscA2*, respectively. As for AtHscB, transient expression analysis of an AtHscA1-YFP fusion protein in tobacco cells showed fluorescence in both mitochondria and cytosol (Figure 2D) as did stable expression of the same fusion protein in transgenic Arabidopsis plants (Figure S1C). Combined this suggest that AtHscB and AtHscA1 may play important roles in both mitochondria and the cytosol in contrast to observations made in yeast where both proteins are exclusively mitochondrial.

### AtHscB can interact with AtIscU1 in mitochondria and in the cytosol

**Figure 3:**
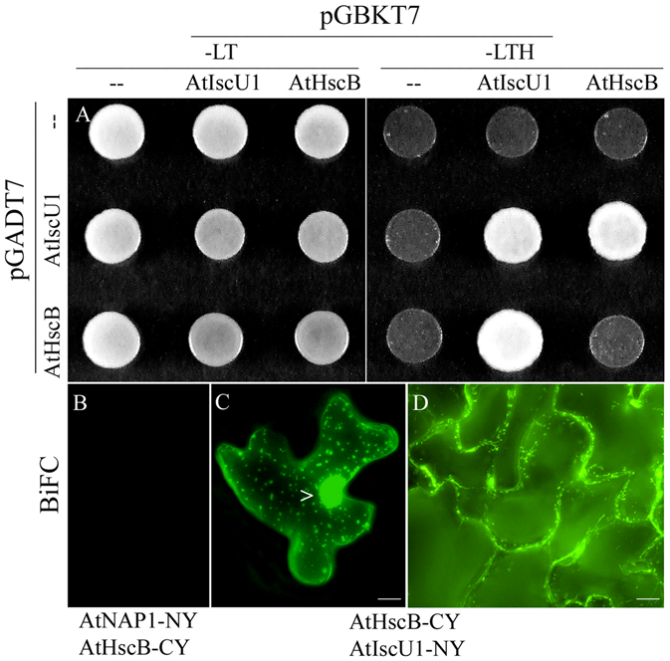
AtHscB interacts with AtIscU1. (A) Yeast two hybrid (YTH). pGADT7 (--), pGBKT7 (--) and pGADT7/pGBKT7 containing either *AtHscB* or *AtIscU*, were transformed in different combinations into yeast HF7c cells and the transformed strains were plated on synthetic dropout medium lacking L-Leucine (Leu) and L-Trypothan (Trp) (-LT). Positive interactions were scored on synthetic dropout medium plates lacking Leu, Trp, and His (L-Histidine) (-LTH). Experiments were performed in triplicate. (B–D) Bimolecular Fluorescence Complementation (BiFC) assays. (B) Tobacco leaves biolistically transformed with pWEN-AtNAP1-NY (N terminal part of YFP) and pWEN-AtHscB-CY (C terminal of YFP) as a negative control. (C) Tobacco leaves biolistically transformed with pWEN-AtIscU1-NY and pWEN-AtHscB-CY showing a fluorescence signal in the mitochondria and the cytosol. Open arrow indicates the nucleus. (D) Mitochondrial and cytosolic fluorescence in Arabidopsis plants transformed with pBA002-AtIscU1-NY (Basta resistant) and pER10-AtHscB-CY (Kan resistant). (Bar = 50 µm).

The scaffold protein IscU/Isu has been shown to play a key role in [Fe-S] biogenesis [48], [49]. To analyse whether AtHscB could interact with AtIscU1, AtIscU2, and AtIscU3 we performed Yeast Two-Hybrid (YTH) and Bimolecular Fluorescence Complementation (BiFC) assays. YTH demonstrated that AtHscB did not interact with AtIscU2 or AtIscU3 but could interact with AtIscU1 and that AtIscU1 could also interact with itself (Figure 3A). Further, transient BiFC experiments in tobacco cells (Figure 3C) and stable BiFC experiments in transgenic Arabidopsis plants (Figure 3D, Figure S1F.) demonstrated clear interaction between AtHscB and AtIscU1 not only in mitochondria but also in the cytosol.

These results were surprising as AtIscU1-GFP fusion experiments have previously shown that AtIscU1 is exclusively localized to mitochondria [40], [41], [50]. Indeed we also show here that an AtIscU1-YFP fusion protein appears exclusively targeted to mitochondria (Figure 2B). However, the dual localization of AtIscU1, when in combination with overexpressed AtHscB in BiFC assays, suggest that AtIscU1 is retained by the high levels of AtHscB in the cytosol through direct protein-protein interactions (Figure 3C, D). In wild-type Arabidopsis, AtHscB is only expressed at low levels which would most probably not allow for sufficient retention of an AtIscU1-YFP fusion protein and hence no detection of AtIscU1-YFP–mediated fluorescence in the cytosol. Combined our data demonstrate that AtHscB can interact with AtIscU1 in both mitochondria and in the cytosol.

### AtHscA1 can complement yeast Ssq1

**Figure 4:**
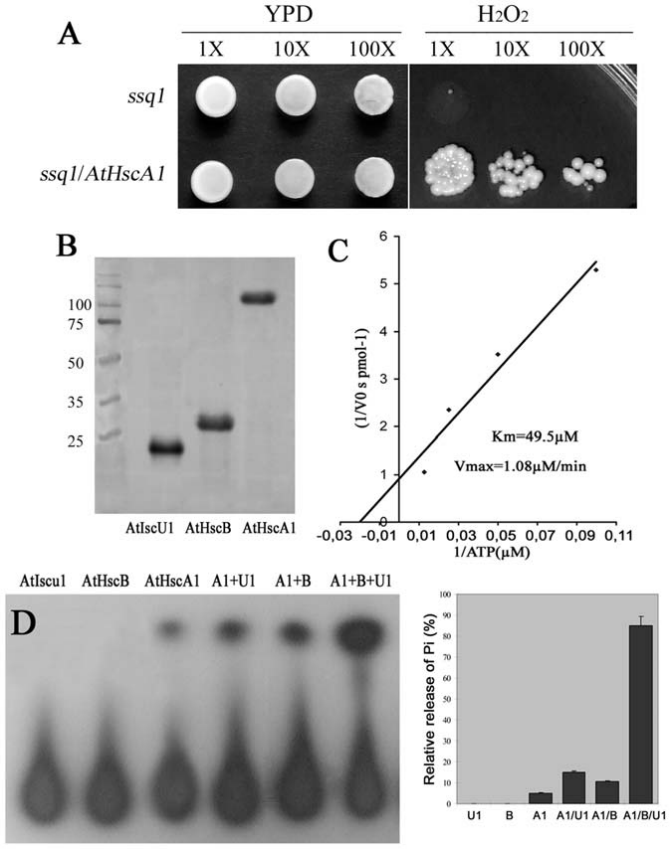
Complementation of the Δ*ssq1* mutant by AtHscA1 and biochemical analysis of AtHscA1. (A) Yeast strains of Δ*ssq1* and Δ*ssq1/*pGADT7-AtHscA1 were cultured overnight in YPD medium and adjusted to similar OD_600_ value. Ten-fold serial dilutions were then spotted on YPD agar plate or on YPD media containing 4 mM H_2_O_2_. (B) Affinity purified AtHscA1, AtHscB and AtIscU1 were verified by SDS/PAGE showing >95% purity. (C) ATPase activity of AtHscA1 showing the Vmax and Km values. (D) The effect of AtHscB and AtIscU1 on the ATPase activity of AtHscA1. Left: An autoradiography image of the reactions; Right: Relative quantification of autoradiography (Triple repeats).

AtHscA1 has high similarity to HscA/Ssq1 of bacteria/yeast (Figure S2). To confirm that AtHscA1 can functionally complement HscA/Ssq1, the *Ssq1* knockout mutant Δ*ssq1* [48] was transformed with pGADT7-AtHscA1 and both strains placed on YPD medium and on YPD containing 4 mM H_2_O_2_ and incubated at 34°C for 4 days (Figure 4A). On YPD medium both Δ*ssq1* and Δ*ssq1*/*AtHscA1* grew well as expected. By contrast, *ssq1* failed to grow on YPD containing H_2_O_2_ whilst Δ*ssq1*/*AtHscA1* showed obvious growth (Figure 4A) demonstrating that AtHscA1 can complement Ssq1.

### AtHscA1 is an ATPase stimulated by AtHscB and AtIscU1

HscA in bacteria and Ssq1 in yeast are both ATPases [49], [51] however, in plants the enzyme activity of AtHscA1 is unknown. We heterologously expressed AtHscA1 in *E. coli* Rosette(DE3)pLysS by auto-induction and affinity purified the protein to >95% purity (Figure 4B). Using γ-P^32^ labeled ATP as a substrate the purified AtHscA1 can clearly hydrolyze ATP (Figure 4D) giving a Km of 49.5 µM and a Vmax of 1.08 µM/min (Figure 4C).

AtHscB and AtIscU1 were also heterologously expressed and purified as for AtHscA1 (Figure 4B). To test the effect of AtHscB or AtIscU1 on AtHscA1 enzyme activity we added purified AtHscB or purified AtIscU1 to AtHscA1 in a 1∶1 stochiometric ratio and performed ATPase assays. From these experiments it was shown that individually AtHscB and AtIscU1 can promote AtHscA1 ATPase activity approximately two-fold. In contrast, when AtHscB, AtIscU1 and AtHscA1 were combined in equal stochiometric concentrations, the ATPase activity of AtHscA1 increased 22-fold (Figure 4D). These experiments clearly demonstrate that AtHscA1 is an ATPase and that in combination AtHscB and AtIscU1 can significantly stimulate AtHscA1-mediated ATP hydrolysis.

### AtHscB expression patterns and AtHscB T-DNA mutant phenotypes

**Figure 5:**
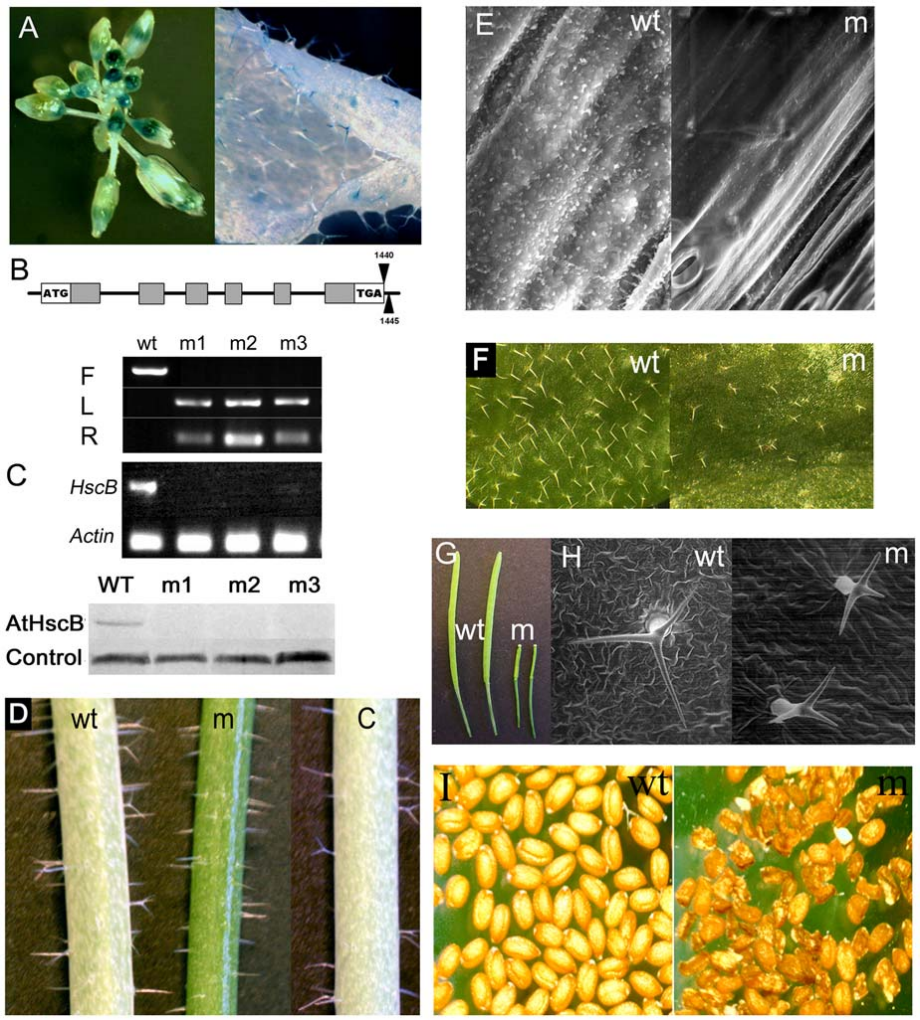
Expression analysis of AtHscB and phenotype of an *AtHscB* T-DNA insertion mutant. (A) GUS expression pattern of *AtHscB* showing high level of expression in anthers and trichomes. (B) Schematic diagram of the *AtHscB* gene structure showing the presence of two T-DNA insertion sites found in the *N585159 AtHscB* T-DNA insertion mutant. Isolation of four *N585159* (AtHscB) homozygous mutants (m1–m4) by PCR analysis. F: PCR using AtHscB-specific primers LP585159/RP585159. L: PCR using primers LP585159/LBb1. LBb1 is T-DNA-specific primer. R: PCR using primers RP585159/LBb1. (C) RT-PCR analysis of *AtHscB* expression in the homozygous T-DNA insertion mutants m1–m4. *HscB*: primers AtHscB-L/R. *Actin*: primers Actin-L/R. Western blot showing the absence of AtHscB in three homozygous mutant plants (m1–m3). (D) Stems of homozygous *N585159* mutants show a waxless phenotype and thus have a smooth bright green appearance compared to wild-type stems. C = homozygous *N585159* mutant plant complemented with a CaMV35S-AtHscB transgene. (E) SEM of wild-type (wt) and *N585159* stems demonstrating much fewer crystals on *N585159* (m) stems than on those of wild-type. Magnification: 5000×. (F) Trichome phenotype of homozygous *N585159* leaves (m) showing smaller and less trichomes than wild-type. Magnification: 10×. (G) Silique development in homozygous *N585159* plants (m) is severely retarded compared to wild-type (wt). (H) SEM of trichomes showing smaller and distorted trichome phenotypes in mutant plants (m) compared to wild-type (wt). Magnification: wt = 184×; m = 244×. (I) Seeds from wild-type (wt) and from homozygous *N585159* (m) plants showing reduced viable seed set in the mutant.

A 1041 nucleotide DNA promoter fragment (1961160–1962200 nt) directly upstream of the AtHscB start codon was PCR-cloned into the β-glucoronidase (GUS) binary vector pBADG and transformed into wild type Arabidopsis. GUS staining of T2 lines showed that AtHscB is universally expressed at low levels but with relatively high levels of expression in anthers and trichomes (Fig. 5A).

An Arabidopsis T-DNA insertion line (SALK_085159) was identified and analyzed by PCR with the T-DNA specific primer LBb1 and the *AtHscB*-specific primers LP585159 and RP585159. Two PCR fragments were obtained and sequenced revealing the presence of two T-DNAs inserted 0 and 5 nt downstream of the *AtHscB* stop codon (Figure 5B). Although the seed set was dramatically reduced in N585159 plants (Figure 5I) several homozygous N585159 plants were isolated (Figure 5B) showing severe down-regulated *AtHscB* transcript and undetectable AtHscB protein (Figure 5C). Phenotypic analysis revealed that homozygous mutants had stems with a shiny bright green appearance, indicating the absence of the epicuticular wax layer (Figure 5D), a similar phenotype to that observed in *CUT1* sense suppressed plants [52]. Indeed, scanning electron microscopy revealed that the stem surface contained much fewer wax crystals than wild type plants (Figure 5E). Homozygous mutants are also conditional sterile, in agreement with the observed reduced seed set, as siliques fail to develop under normal growth conditions whilst in a humid environment siliques develop as in wild-type (Figure 5G, Figure S3A). These data suggests that AtHscB-deficiency results in reduced or diminished very-long-chain fatty acids (VLCFAs) biosynthesis. To confirm that the observed mutant phenotypes were caused by AtHscB-deficiency, the homozygous mutant was transformed with wild-type *AtHscB* followed by phenotypic characterization. More than 95% (20 out of 21) of transformed resistant plants showed a wild type phenotype (Figure 5D, Figure S3B, 3C) confirming that *AtHscB*-deficiency is responsible for the observed mutant phenotypes. Due to the high level of *AtHscB* expression in trichomes (Figure 5A), we monitored trichome development in homozygous N585159 mutant plants. In agreement with the *AtHscB* gene expression patterns homozygous mutants not only have fewer trichomes than wild-type (Figure 5F) but these were also smaller in size and often distorted (Figure 5H).

**Figure 6:**
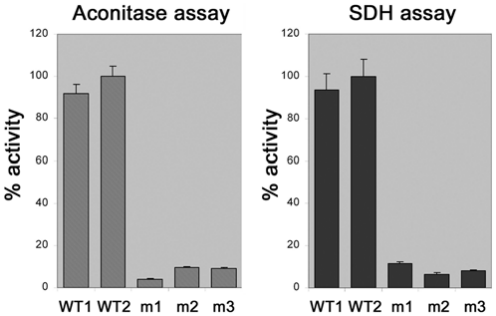
Aconitase and succinate dehydrogenase (SDH) enzyme assays of wt and homozygous *N585159* plants (Four repeats). Two wild-type (WT1 and WT2) plants are compared to three homozygous *N585159* mutant plants (m1, m2 and m3).

To test whether AtHscB indeed has an effect on iron sulfur proteins in Arabidopsis, we assayed both aconitase and succinate dehydrogenase (SDH) activities in wild type plants and in homozygous N585159 mutant plants. Figure 6 shows that in the homozygous mutants both aconitase and SDH enzyme activities are reduced to approximately 10% of wild-type levels. These data, combined with other finding shown in this study, support the notion that AtHscB has a role in iron sulfur protein biogenesis in Arabidopsis.

## Discussion

In this study we provide data suggesting that the Jac1-homolog AtHscB is involved in iron sulfur protein biogenesis in Arabidopsis. Several points of evidence demonstrates that AtHscB is indeed a Jac1-like protein: (i) AtHscB can rescue the yeast Δ*jac1* mutant (Figure 1), (ii) AtHscB together with AtIscU1 can significantly enhance the ATPase activity of the HscA/Ssq1-like protein AtHscA1 (Figure 4) and (iii) Diminished levels of AtHscB in mutant plants severely reduces the enzyme activities of aconitase and succinate dehydrogenase, two important iron sulfur proteins found in mitochondria (Figure 6). Aconitase in yeast seems to have a dual function as it has an influence on the glyoxylate shunt in the cytosol and the TCA cycle in mitochondria, corroborating our finding that aconitase activity is highly diminished in an AtHscB mutant, lacking AtHscB in both the cytosol and mitochondria.

Because of the clear relationship between AtHscB and AtHscA1 in Arabidopsis we also demonstrated, through yeast complementation experiments, that AtHscA1 is an HscA/Ssq1-like protein (Figure 4 and Figure S2). Arabidopsis contains a second HscA/Ssq1-like protein (At5g09590), AtHscA2, and it will be interesting to examine its connection with AtHscB and its possible role in iron-sulfur protein biogenesis (Figure S2).

Surprisingly, both AtHscB and AtHscA1 show dual localization where they are present in both mitochondria and the cytosol as revealed by YFP fusion protein localization studies and immunogold labeling experiment (Figure 2 and Sup. Figure 1B–E). The fact that immunogold experiments demonstrate that endogenous AtHscB is present in both mitochondria and the cytosol eliminates the dual localization being due to overexpression of the transgene. Many dual localized proteins have been shown to have a low MitoProtII score and the AtHscB mitochondrial targeting sequence has a low MitoProtII value (http://ihg.gsf.de/ihg/mitoprot.html). Based on our findings it is reasonable to suggest that AtHscB, AtHscA1 and AtIscU1 may function in both mitochondria, and through retention of AtIscU1 by AtHscB (Figure 3), in the cytosol. The dual localization of the AtHscB/AtHscA1/AtIscU protein cluster suggests spatial coordination of [Fe-S] biogenesis in plants between subcellular compartments which has been suggested for sulfur acquisition within the ISC system [42]. The fact that AtHscB possibly retains AtIscU in the cytosol implies that AtHscB may act as a control point for the balance of AtHscB/AtHscA1/AtIscU1-mediated [Fe-S] biogenesis in Arabidopsis. Based on the data presented AtIscU1 is only detected in the cytosol when AtHscB accumulates and it will be interesting to examine cytosolic retention of AtIscU1 during plant development and during conditions that favor cytosolic [Fe-S] biogenesis. Progress has been made on the biogenesis of cytosolic [Fe-S] [43] where AtNBP35 plays a key role in this process. How AtNBP35 relates to AtHscB/AtHscA1/AtIscU1 in the cytosol remains an exciting challenge.

Based on the evolutionary conservation of AtHscB and AtHscA1 it is reasonable to assume that the interaction of AtIscU1 with AtHscA1 is regulated by AtHscB as seen in bacteria and we have shown here that AtHscB stimulates the ATPase activity of the AtHscA1/AtIscU1 complex (Figure 4D). However, the molecular mechanism and function of AtHscB-mediated enhancement of AtHscA1 ATPase activity is unknown but may contribute to substrate specificity.

The fact that AtHscB/AtHscA1/AtIscU1 are localized to the cytosol and mitochondria may indicate some form of crosstalk between [Fe-S] biogenesis systems in these two spatially separated subcellular compartments. Such crosstalk would be essential in light of the importance of maintaining iron and sulfur homeostasis.

Iron-sulfur proteins are involved in many fundamental and diverse biological processes dictating that the consequences of inappropriate [Fe-S] biogenesis may have dramatic and pleotrophic effects on plants. Indeed several reports have shown severe developmental defects in Arabidopsis in response to inappropriate [Fe-S] biogenesis such as embryo lethality in both AtSufE and AtSufC loss-of-function mutants [26], [27]. AtHscB mutant plants have dramatically reduced seed set (Figure 5I) and this is in good agreement with previous studies showing that reduced SDH activity leads to altered gametophytic development [53]. However and somewhat surprisingly, dramatically reduced levels of AtHscB also results in relatively specific phenotypic consequences. The molecular basis for the waxless phenotype (decreased wax crystals and conditional sterility) of the homozygous AtHscB mutant (Figure 5E) is intriguing and links iron-sulfur protein biogenesis to VLCFA biosynthesis in Arabidopsis. The role of AtHscB in VLCFA biosynthesis is unclear however VLCFA biosynthesis most probably involves [Fe-S] enzymes that are compromised in plants lacking AtHscB. Indeed it will be interesting to investigate VLCFA biosynthetic enzymes in terms of [Fe-S] content. Similarly, the altered trichome structure in AtHscB mutant plants, accompanied by the fact that *AtHscB* expression occurs in these cells (Figure 5A), suggests that [Fe-S] biogenesis is important for this specialized process.

The intriguing phenotypic effects of AtHscB deficiency on plant development and the unexpected dual localization of both AtHscB and AtHscA1, highlights the complex nature of [Fe-S] biogenesis in Arabidopsis. It further implies that although iron-sulfur protein biogenesis represents a fundamental biological process, proteins involved in [Fe-S] biogenesis are also highly specific in terms of controlling defined developmental processes with seemingly intricate regulatory circuits.

## Materials and Methods

### Plants and growth conditions

Wild-type Arabidopsis, transgenic Arabidopsis and the *AtHscB* T-DNA insertion mutant Salk_085159 (N585159) were grown at 21°C with 16 h of light (100 µmol photons m^–2^ s^–1^) per day and 60% humidity unless otherwise stated. The *AtHscB* T-DNA insertion mutant Salk_085159 (N585159) [54] was obtained from NASC (European Arabidopsis Stock Centre, Nottingham).

### Plasmids and primers

A GFP-GUS DNA fragment was PCR amplified using primers GFPGUS-L/GFPGUS-R (Primer sequences in this study can be found in Table S1) and pBADG was constructed by inserting GFP-GUS into *Pac*1 and *Sac*I of pBA002a [55]. A 1041 nt fragment, directly upstream of the *AtHscB* ATG was amplified with primers P06410-AscI-L/P06410-PacI-R, cloned into *Asc*I and *Pac*I of pBADG and transformed into wild-type Arabidopsis for GUS expression analysis. *AtHscB* and *AtIscU1* were PCR amplified using primers AtHscB-GBK-L/AtHscB-GBK-R and AtIscU1-GBK-L/AtIscU1-GBK-R, and cloned into *Nde*I and *Bam*HI of pGBKT7 or pGADT7 (Clontech). *AtHscB*, *AtIscU1* and *AtHscA1* were amplified using primers AtHscB-XhoI-L/AtHscB-KpnI-R, AtIscU1-XhoI-L/AtIscU1-KpnI-R and AtHscA1-KpnI-L/AtHscA1-KpnI-L R, digested with *Xho*I/*Kpn*I or with *Kpn*I and inserted into pWEN18, pWEN-N-YFP (YFP amino acids 1–154) and pWEN-C-YFP (YFP amino acids 155–238). *AtHscB*, *AtIscU1*, and *AtHscA1* were PCR cloned into pET28a using primers AtHscB-ET-L/AtHscB-ET-R AtIscU1-ET-L/AtIscU1-ET-R and AtHscA1-ET-L/AtHscA1-ET-R. *AtHscB* was PCR amplified with primers AtHscB-XhoI-L/AtHscB-SpeI-R, digested with *Xho*I/*Spe*I and cloned into pBA002 for N585159 complementation experiments. Primers are shown in Table S1.

### Subcellular localization and BiFC assays

pWEN18, pWEN-N-YFP (pWEN-NY) and pWEN-C-YFP (pWEN-CY) containing *AtHscB*, *AtIscU1* and *AtHscA1* were transiently expressed in tobacco cells. To test for AtHscB and AtIscU1 interactions pWEN-AtHscB-NY/pWEN-AtIscU1-CY or pWEN-AtHscB-CY/pWEN-AtIscU1-NY were used to bombard and transiently expressed in tobacco leaves. For stable expression analysis, *AtHscB*, *AtHscA1* and *AtIscU1* fused to YFP, NY and CY, were cloned into *Asc*I/*Pac*I of pBA002 and pER10 and transformed into Arabidopsis [56]. All fluorescence analysis was performed on a Nikon TE-2000U inverted microscope (Nikon, Japan) and Volocity II software (Improvision, UK). For electron microscopy/immunogold analysis standard protocols were followed using a JEOL 1220 (Electron Microscopy Laboratory, University of Leicester, UK) and 17 images analyzed. Pre-immune serum was used as a control. An AtHscB polyclonal antibody was generated following standard protocols as instructed by Harlan Laboratories.

### Yeast Transformation

pGBKT7 and pGADT7 containing *AtHscB* and *AtIscU1* and control vector controls were transformed into HF7c and tested for His auxotrophy restoration following the Matchmaker two-hybrid system III manual (Clontech).

For Δ*jac1* complementation, Δ*jac1*-pRS316-Jac1 was transformed with pGBKT7-AtHscB or pGADT7-AtHscB and screened on SD/-Trp (minus L-trypothan) or SD/-Leu (minus L-leucine). Colonies were streaked onto 5-FOA media (YPD media, 1.0 mg/ml 5-Fluoroorotic Acid, 0.05 mg/ml uracil) to screen for complementation. Isolated colonies were verified by RT-PCR using primers Jac1-L/R and AtHscB-L/R.

For Δ*ssq1* complementation, Δ*ssq1* was transformed with pGBKT7-AtHscA1 and positive transformants screened on SD/-Trp. Overnight cultures were spotted onto YPD or H_2_O_2_ plates (YPD, 4 mM H_2_O_2_) and incubated at 34°C for 4 days.

### Protein expression and purification

pET28a containing *AtHscB*, *AtIscU1* and *AtHscA1* were transformed into *E. coli* Rosetta(DE3)pLysS (Novagen) and expression performed for 20 h in auto-induction ZYM-5052 media [57]. Cells were disrupted in 50 mM Tris–HCl (pH 8.0), 25% sucrose, 5 mM MgCl_2_, 100 mM NaCl, 1% Triton X-100 and 10 µM/ml Benzonase^®^ Nuclease (Novagen) and the proteins purified using TALON affinity resin (BD Biosciences). Purity was assessed by SDS–PAGE analysis.

#### Enzyme assays

ATPase assays were performed as described previously [25], [26]. Each reaction mixture contained 50 mM Tris-HCl (pH 7.4), 50 mM NaCl, 0.1 mM EDTA, 1.5 mM dithiothreitol, 10 mM KCl. Each assay was performed using 2 µM purified protein and 0.1 µCi/µl [γ-^32^P] ATP (1 Ci = 37 GBq) (specific activity 10 mCi/mmol). All reactions were terminated using 1 µl of 1 M formic acid. Samples were applied to TLC plates (Macherey and Nagel), developed in 0.5 M LiCl and 0.5 M formic acid buffer, and visualized by autoradiography. The hydrolyzed ATP was quantified using phosphorimager.

Aconitase and succinate dehydrogenase (SDH) assays were performed following Stehling *et al* [58]. Aconitase assay: A sample cuvette containing 950 µl aconitase buffer (100 mM Trienthanolamine pH 8.0, 1.5 mM MgCl_2_, 0.1% Triton X-100), 200 µM *cis*-aconitate, 1.3 mM NADP+, 400 µU IDH (isocitrate dehydrogenase), and a reference cuvette lacking *cis*-aconitate and IDH was prepared. To each cuvette, protein extraction (≈40 µg protein) was added and absorption increase at 340 nm in a double-beam spectrophotometer was measured (ε_340nm_ = 6220 M^−1^ cm^−1^). SDH assay: Two cuvettes containing 950 µl SDH buffer (50 mM Tris-H_2_SO_4_ pH 7.4, 0.1 mM EDTA, 1 mM KCN, 0.1% Triton X-100), 0.25% succinate, 70 µM dichlorophenol-indophenol, and 60 µM decylubiquinone were prepared. 0.25% malonate was added to the reference cuvette and the assay was initated by adding cell suspension (≈40 µg protein) to the second cuvette. The absorption decrease was measured at 600 nm in a double-beam spectrometer (ε_600nm_ = 21,000 M^−1^ cm^−1^).

## Supporting Information

Table S1 Primers used in this study
(0.04 MB DOC)

Figure S1 A. Amino acid sequence alignment of AtHscB with HscB of E.coli and Jac1 of yeast. Accession number of HscB is NP_417022 and Jac1 NP_011497. Filled arrow indicates the possible cleavage site of the signal peptide. * underlines the important motif conserved for all the three proteins. B. Stable expression of AtHscB-YFP in Arabidopsis. C. Stable expression of AtHscA1-YFP in Arabidopsis. D. Western blot showing the specificity of the AtHscB antibody. Lane a) represents wild-type E. coli cell extract, lane b) represents cell extract from E. coli expressing AtHscB and lane c) represents total cell extract from wild-type Arabidopsis. The slight size increase in lane b) is due to the presence of a 6xHis affinity tag on the protein. E. Immunogold labelling using pre-immune serum. F. Western blot to check the AtHscB expression in AtHscB-CY (C terminal of EYFP)/AtIscU-NY (N terminal of EYFP) double transformed plants. WT. wild type plants. OX-1 and OX-2 are two double transformed plants. The upper panel used anti-AtHscB. The lower panel is a loading control stained with coomassie.
(9.04 MB TIF)

Figure S2 Amino acid sequence alignment of HscA-like proteins. Accession number: HscA of Ecoli, NP_417021; Ssq1 of yeast, NP_013473; At4g37910 (AtHscA1), NP_195504; At5g09590 (AtHscA2), NP_196521.
(4.85 MB TIF)

Figure S3 A. Conditional sterile phenotype of N585159. Mut (humid): mutant grown under humid condition; Mut: mutant grown under normal condition; Wild-type plant as positive control. Inserted icon: Siliques of WT (wild-type), Mut(h) (mutant plants grown under humid condition) and Mut (mutant plants grown under normal condition). B. SEM of N585159 C. SEM of N585159 complemented with AtHscB.
(5.79 MB TIF)
