# Supplementary material for: Dual Localized AtHscB Involved in Iron Sulfur Protein Biogenesis in Arabidopsis
Source: PLoS One. 2009 Oct 29;4(10):e7662. doi: 10.1371/journal.pone.0007662 (PMC2764847; doi:10.1371/journal.pone.0007662)
Supplement: Table S1 — Primers used in this study (0.04 MB DOC) [file pone.0007662.s001.doc]

**Table S1.**

| Primer name | Sequence | Restriction site (underlined) |
| --- | --- | --- |
| LP585159  RP585159  LBb1 | 5’ GACATTAACAAAGCGGCTGTC 3’  5’ GTGGGAATTCAGTCATTCACC 3’  5’ GCGTGGACCGCTTGCTGCAACT 3’ |  |
| AtHscB-GBK-L  AtHscB-GBK-R | 5’ ATCATATGATGAAGAAAACGAAGACGATGG 3’  5’ ATGGATCCTCATAGCTTCTTCAAAATTTCTTCG 3’ | NdeI  BamHI |
| AtIscU1-GBK-L  AtIscU1-GBK-R | 5’ATCATATGATGATGCTCAAGCAAGCTC 3’  5’ATGGATCCTCAAGCCTGTGTGGTTTCTC 3’ | NdeI  BamHI |
| AtHscB-ET-L  AtHscB-ET-R | 5’ ATGGATCCATGAAGAAAACGAAGACGATGG 3’  5’ ATCTCGAGTAGCTTCTTCAAAATTTCTTCG 3’ | BamHI  XhoI |
| AtIscU1-ET-L  AtIscU1-ET-R | 5’ ATGGATCCATGATGCTCAAGCAAGCTG 3’  5’ ATCTCGAGAGCCTGTGTGGTTTCTC 3’ | BamHI  XhoI |
| AtHscA1-ET-L  AtHscA1-ET-R | 5’ ATGGATCCATGGCCTCCTGTCGCGCTTCTAC 3’  5’ ATGAGCTCCTTCCTTGAACCACTGGCCTC 3’ | BamHI  SacI |
| AtHscB-XhoI-L  AtHscB-KpnI-R | 5’ AATCTCGAGATGAAGAAAACGAAGACGATGG 3’  5’ AATGGTACCTAGCTTCTTCAAAATTTCTTCGCAG 3’ | XhoI  KpnI |
| AtIscU1-XhoI-L  AtIscU1-KpnI-R | 5’ ATTCTCGAGATGATGCTCAAGCAAGCTG 3’  5’ ATTGGTACCAGCCTGTGTGGTTTCTCCTG 3’ | XhoI  KpnI |
| AtHscA1-XhoI-L  AtHscA1-KpnI-R | 5’ ATGGTACCATGGCCTCCGTCGCGCTTCTAC 3’  5’ ATGGTACCCTTCCTTGAACCACTGGCCTC 3’ | KpnI  KpnI |
| Jac1-L  Jac1-R | 5’ TTTACGAGCTGTTCCCAAAGA 3’  5’ TTTCCAATTGTTTTCCTGGAG 3’ |  |
| Ssc1-L  Ssc1-R | 5’ GTTGTTGCCGTTTTCGATTT 3’  5’ AACCTCACCGGACAAGACAG 3’ |  |
| GFPGUS-L  GFPGUS-R | 5’ GGGCGCGCCTCGAGTTAATTAAGATATCCCGCGGATGGT 3’  5’ ATGAGCTCTCATTGTTTGCCTCCCTGCT 3’ | AscI-PacI  SacI |
| P06410-AscI-L  P06410-PacI-R | 5’ AATGGCGCGCCTGAAGCCTTTTTCTTATTCCCC 3’  5’ AATTTAATTAATGGAGAGATCGTCGTGTATCG 3’ | AscI  PacI |
| AtHscB-SpeI-R | 5’ AATACTAGTTAGCTTCTTCAAAATTTCTTCG 3’ | SpeI |
| AtHscB-L  AtHscB-R | 5’ ATGAAGAAAACGAAGACGATGG 3’  5’ TCATAGCTTCTTCAAAATTTCT 3’ |  |
| Actin-L  Actin-R | 5’ AGCTCCGTATTGCTCCTGAA 3’  5’ TGGTTTTCGAGGTCTCCATC 3’ |  |
