# Supplementary figures and images for: Dual Localized AtHscB Involved in Iron Sulfur Protein Biogenesis in Arabidopsis
Source: PLoS One. 2009 Oct 29;4(10):e7662. doi: 10.1371/journal.pone.0007662 (PMC2764847; doi:10.1371/journal.pone.0007662)

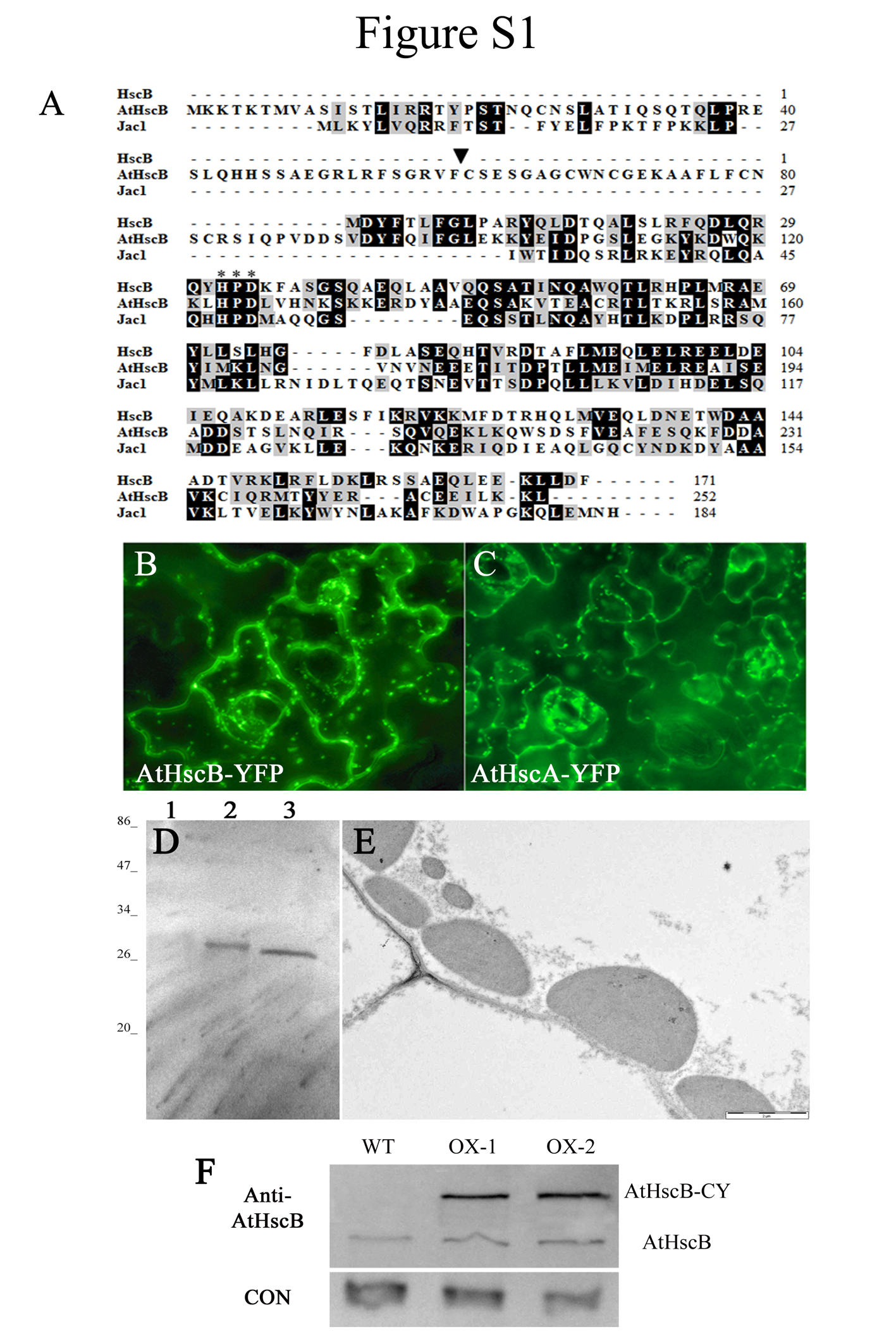

Supplement: Figure S1 — A. Amino acid sequence alignment of AtHscB with HscB of E.coli and Jac1 of yeast. Accession number of HscB is NP_417022 and Jac1 NP_011497. Filled arrow indicates the possible cleavage site of the signal peptide. * underlines the important motif conserved for all the three proteins. B. Stable expression of AtHscB-YFP in Arabidopsis. C. Stable expression of AtHscA1-YFP in Arabidopsis. D. Western blot showing the specificity of the AtHscB antibody. Lane a) represents wild-type E. coli cell extract, lane b) represents cell extract from E. coli expressing AtHscB and lane c) represents total cell extract from wild-type Arabidopsis. The slight size increase in lane b) is due to the presence of a 6xHis affinity tag on the protein. E. Immunogold labelling using pre-immune serum. F. Western blot to check the AtHscB expression in AtHscB-CY (C terminal of EYFP)/AtIscU-NY (N terminal of EYFP) double transformed plants. WT. wild type plants. OX-1 and OX-2 are two double transformed plants. The upper panel used anti-AtHscB. The lower panel is a loading control stained with coomassie. (9.04 MB TIF) [file pone.0007662.s002.tif]

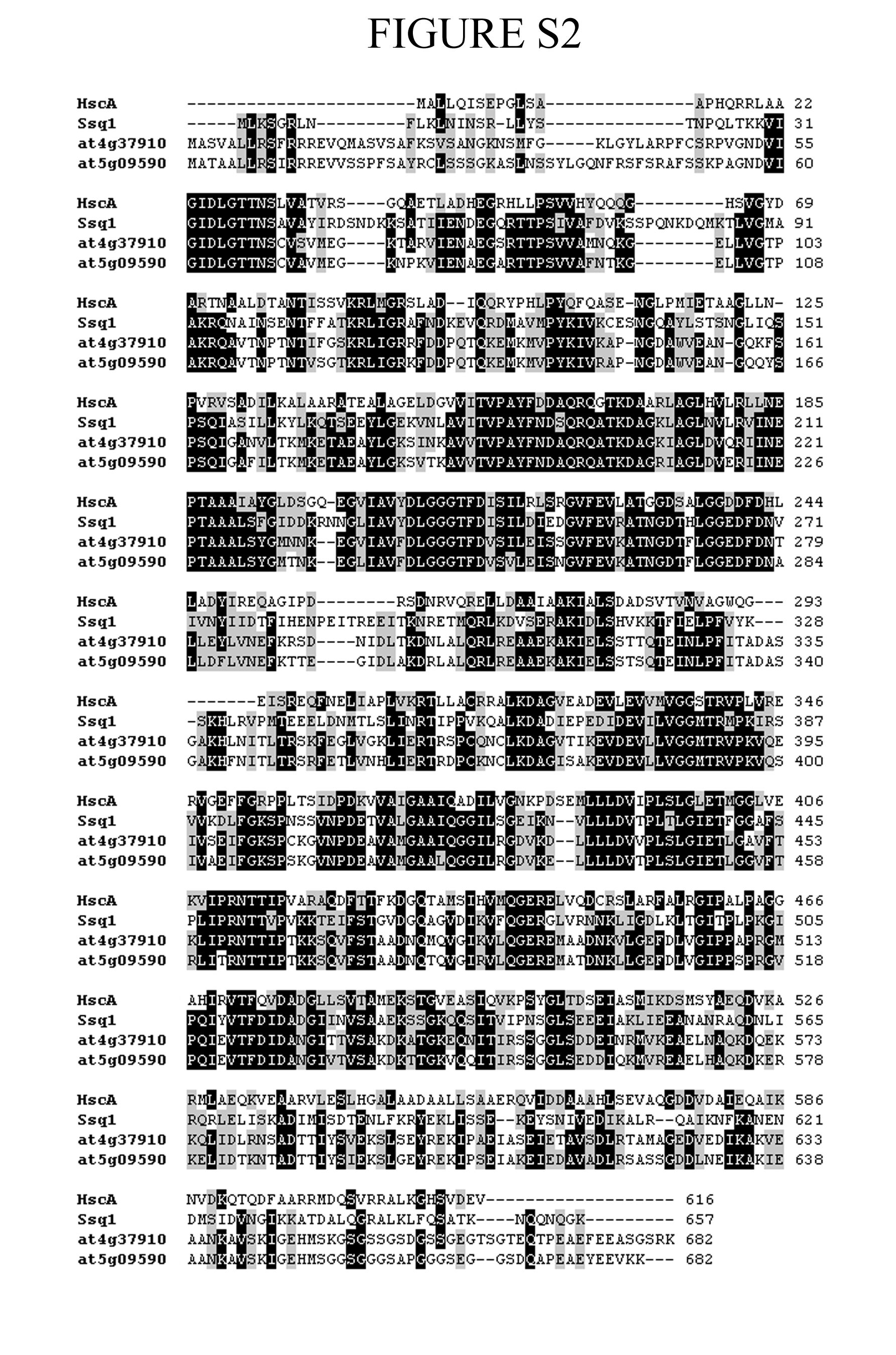

Supplement: Figure S2 — Amino acid sequence alignment of HscA-like proteins. Accession number: HscA of Ecoli, NP_417021; Ssq1 of yeast, NP_013473; At4g37910 (AtHscA1), NP_195504; At5g09590 (AtHscA2), NP_196521. (4.85 MB TIF) [file pone.0007662.s003.tif]

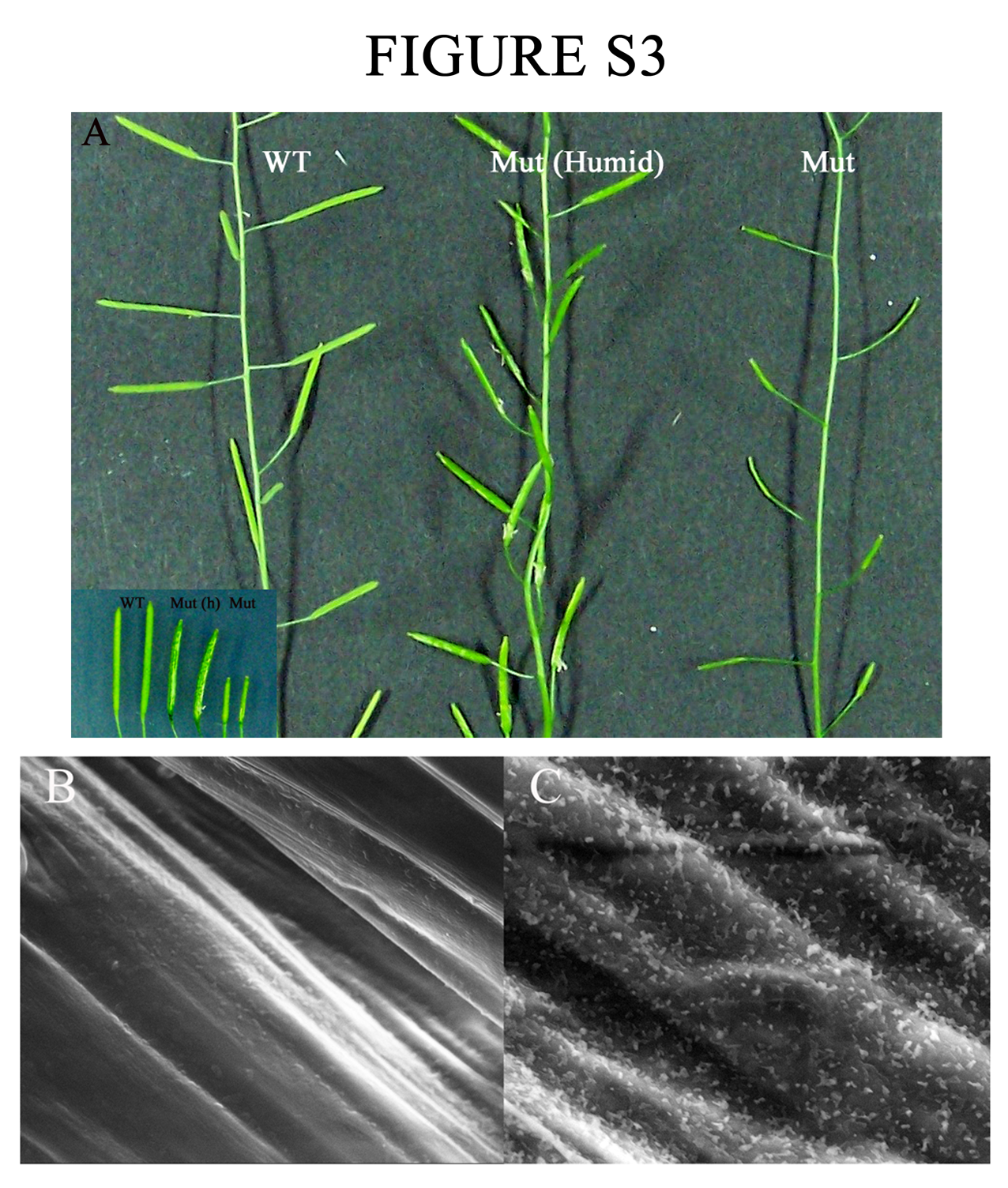

Supplement: Figure S3 — A. Conditional sterile phenotype of N585159. Mut (humid): mutant grown under humid condition; Mut: mutant grown under normal condition; Wild-type plant as positive control. Inserted icon: Siliques of WT (wild-type), Mut(h) (mutant plants grown under humid condition) and Mut (mutant plants grown under normal condition). B. SEM of N585159 C. SEM of N585159 complemented with AtHscB. (5.79 MB TIF) [file pone.0007662.s004.tif]
